# Distribution of new graphic warning labels: Are tobacco companies following regulations?

**DOI:** 10.1186/1617-9625-5-14

**Published:** 2009-08-25

**Authors:** Nick Wilson, Jo Peace, Judy Li, Richard Edwards, Janet Hoek, James Stanley, George Thomson

**Affiliations:** 1 Department of Public Health, University of Otago, Wellington, New Zealand; 2 Department of Marketing, University of Otago, Dunedin, New Zealand

## Abstract

**Objective:**

To test the hypothesis that tobacco companies would not follow a regulation that required seven new graphic health warnings (GHWs) to be evenly distributed on cigarette packs and that they would distribute fewer packs featuring warnings regarded by smokers as being more disturbing.

**Methods:**

Cross-sectional survey of purchased packs (n = 168) and street-collected discarded packs (convenience sample of New Zealand cities and towns, n = 1208 packs) with statistical analysis of seven types of new GHWs. *A priori *warning impact was judged using three criteria, which were tested against data from depth interviews with retailers.

**Results:**

The GHWs on the purchased packs and street-collected packs both showed a distribution pattern that was generally consistent with the hypothesis ie, there were disproportionately more packs featuring images judged as "least disturbing" and disproportionately fewer of those with warnings judged "more disturbing". The overall patterns were statistically significant, suggesting an unequal frequency of the different warnings for both purchased (p < 0.0001) and street-collected packs (p = 0.035). One of the least disturbing images (of a "corpse with toe-tag") dominated the distribution in both samples. Further analysis of the street-collected packs revealed that this image appeared disproportionately more frequently on manufactured cigarettes made by each of the three largest New Zealand tobacco companies. Although stock clustering could explain the purchase pack result, there were no obvious reasons why the same uneven warning distribution was also evident among the street-collected packs.

**Conclusion:**

These results suggest that tobacco companies are not following the regulations, which requires even distribution of the seven different GHWs on cigarette packs; further monitoring is required to estimate the extent of this non-compliance. As an immediate measure, governments should strictly enforce all regulations applying to health warnings, particularly given that these are an effective tobacco control intervention that cost tax payers nothing.

## Background

In response to the Framework Convention on Tobacco Control, New Zealand upgraded its requirements for health warnings and introduced new regulations under the Smoke-free Environments Act [1]. These new regulations require tobacco companies to replace text-based health warnings with graphic health warnings (GHWs).

The first set of warnings was phased in from early 2008, and by the end of August 2008, all tobacco packets sold in New Zealand were required to have one of seven GHWs displayed on the front and back of the packet. Tobacco companies opposed the introduction of GHWs, arguing that these would: "denigrate or shame adult consumers" and "denigrate the pack and demonise the tobacco industry" [2]. These comments raise questions about their willingness to comply with both the letter and the spirit of the regulations, which require them to ensure that: "each warning message, corresponding explanatory message, and corresponding graphic appears as nearly as possible on an equal number of retail packages of each different brand of cigarettes or loose or pipe tobacco" [1]. We therefore tested the hypothesis that tobacco companies would not follow this regulation, and would instead distribute fewer of the packs featuring warnings regarded by smokers as being more disturbing.

## Methods

The new cigarette packs were required to have one of seven images (ie, the first seven pictures of those shown on the New Zealand Ministry of Health website [3]). To assess how smokers would interpret these seven GHWs, and which they would see as most and least disturbing, we made *a priori *judgements based on: the specificity with which the image depicted smoking harms; the proportion of smokers who might perceive the image as personally relevant; and the visibility of the harm depicted (damage visible externally versus damage to internal organs).

Following this assessment, the "corpse with toe-tag" image was judged to be "least disturbing", since the causal link with smoking was less apparent. Images of damaged internal organs (ie, the heart and the lung images) were assumed to have an intermediate impact as they are relevant to all smokers, but depict less visible impacts. The image of a "pregnant woman and infant" was also judged to have an intermediate impact for most smokers, since the warning applied directly to only a small proportion of them. Images of external body parts were regarded as likely to be "most disturbing", as these may apply to all smokers and are highly public (ie, the diseased mouth, eye and gangrenous toes). These hypothesised responses were generally consistent with research testing different labels undertaken by one of us (JH). This research found that the more visually impactful the warning (as assessed using retained factors based on paired antonyms scored on semantic differential scales), the higher the level of fear elicited and the stronger the likelihood of cessation-related behaviours [4].

To further test these criteria, we compared them with data obtained from a separate unpublished study that interviewed Wellington shopkeepers (undertaken by one of us (JL)). Shopkeepers who noticed customers' preferences for different packs (n = 17/21), indicated that customers least preferred warnings featuring the "diseased mouth/mouth cancer" (n = 15), the "eye/blindness" (n = 9), and "gangrenous toes" (n = 8). Shopkeepers reported that their customers were more likely to request images depicting a "pregnant woman and infant" (n = 7) or a "corpse with toe-tag" (n = 5).

We initially collected data by reviewing the warnings on 168 packs of the most popular brands, purchased to contribute to an international tobacco products repository [5]. The packets were purchased from three supermarkets in Wellington and Wairarapa in October 2008, using a standard procedure [5]. As there is a possibility that stores could receive print runs of the same pack design, thus resulting in clustering of the GHWs, we also collected a sample of discarded manufactured cigarette packs, which should not be prone to any print run effects. We undertook convenience sampling in four cities and six New Zealand towns/rural locations during November 2008 to January 2009. Collection was undertaken by some of the authors (n = 4), colleagues (n = 9), and a paid student (with all of the collectors other than the authors being unaware of the precise purpose of the study). All packs observed in the street were collected, no matter how damaged these were and regardless of whether they featured a GHW (ie, we included overseas and pre-graphic warning packs).

To analyse the distribution of the pack images we used a multinomial logistic regression model with no predictors (using PROC LOGISTIC in SAS 9.1; SAS Institute Inc., N.C.). This allowed calculation of an overall chi-square value to test whether equal proportions of the different pack images were collected, as well as odds for each image relative to the reference category (for which we used the "corpse with toe-tag" image based on our judgement that it would be least disturbing for smokers). Odds values and a 95% confidence interval (CI) for each non-reference picture type were calculated (ie, so that an odds value of < 1 indicated that a particular image was less common than the "corpse with toe-tag" image).

## Results

There were 1208 packs with a New Zealand GHW found in the street collection. Table 1 shows the warning distribution pattern observed on the purchased and street-collected packs. A chi-square test across all GHWs indicated that these were not distributed evenly across the different packs in the purchased dataset (X^2 ^[6 df] = 32.67, p < 0.0001). A similar test for the street-collected packs was also significant (X^2 ^[6 df] = 13.59, p = 0.035). The pattern of distribution of GHWs was consistent with the hypothesis that disproportionately more of the "corpse with toe-tag" warning would be evident on both the purchased and street-collected packs. Following our *a priori *classification, we used this warning as a reference and compared the prevalence of other warnings against it. These analyses revealed statistically significant differences in the odds relative to nearly all other warnings.

**Table 1:** Distribution of the seven required graphic health warnings in purchased packs and street-collected packs

**Theme of the graphic warning**	***A priori *assessment of the images**	**Purchased packs (collected October 2008)**			Street-collected packs* (collected November 2008 to January 2009)		
		**Observed (N)**	**Percentage**	Odds**	**Observed (N)**	**Percentage**	Odds**
Corpse with toe-tag	Least disturbing	43	25.6%	*Reference category*	215	17.8%	*Reference category*
Pregnant woman & infant	Moderately disturbing	25	14.9%	0.58 (0.36, 0.95)	169	14.0%	0.79 (0.64, 0.96)
Damaged heart	Moderately disturbing	36	21.4%	0.84 (0.54, 1.3)	158	13.1%	0.73 (0.6, 0.9)
Damaged lungs	Moderately disturbing	17	10.1%	0.40 (0.23, 0.69)	174	14.4%	0.81 (0.66, 0.99)
Gangrenous toes	Most disturbing	17	10.1%	0.40 (0.23, 0.69)	156	12.9%	0.73 (0.59, 0.89)
Eye (blindness)	Most disturbing	18	10.7%	0.42 (0.24, 0.73)	169	14.0%	0.79 (0.64, 0.96)
Diseased mouth/cancer	Most disturbing	12	7.1%	0.28 (0.15, 0.53)	167	13.8%	0.78 (0.63, 0.95)

**Total**		**168**			**1208**		

* For those packs that had the seven required GHWs (ie, excluding NZ packs with text-only warnings (n = 8), overseas made packs (n = 42), and also NZ packs showing the next batch of seven GHWs that began to appear in January 2009 (n = 52).

** This column presents the odds of being the specified image rather than the least disturbing image (ie, odds < 1 indicate that the image was less common than the "corpse with toe-tag" image).

Further analysis of street-collected packs indicated that the "corpse with toe-tag" image was the commonest image used on the three largest New Zealand tobacco companies' products (at 16.0%, 22.9% and 24.5%). Table 1 also shows that the least commonly detected warnings for both purchased and street-collected packs, were in the category our *a priori *classification identified as "most disturbing". This was particularly evident from the purchased packs sample.

## Discussion

The purchased pack results in this study showing uneven GHW distribution may have been a chance finding (as a result of batch clustering). However, the street-collected packs suggest that tobacco companies operating in New Zealand are not following the requirements of the new regulations, which specify an even distribution of GHWs on cigarette packs. Such non-compliance would be consistent with the tobacco companies' stated opposition to GHWs in New Zealand. It would also be consistent with their opposition to other tobacco control interventions in New Zealand over past decades [6].

While it is *plausible *that the more disturbing warnings were the first to be employed, and were featured more on packs distributed and sold earlier in 2008 (and thus there was a balance throughout the required 12-month period as stated in the regulations), this would seem unlikely for several reasons. First, featuring the most disturbing images as soon as GHWs were required would be counter to the tobacco industry's over-riding mandate to maximise profit. Second, featuring the most disturbing warnings first would be inconsistent with the industry's opposition to measures they argued would "shame" smokers. Finally, although it is also conceivable that smokers might dispose of the more disturbing packs differently (eg, into rubbish bins rather than dropping them on the street) this would also seem unlikely, as it would require a higher level of conscious thought than is typically associated with littering.

It is also conceivable that the distribution of street-collected packs reflects purchase of particular packs. But for this to result in a distorted distribution of packs for the whole smoker population, retailers who ran low of stock with particular "favoured" warnings would have to selectively re-order packs featuring these warnings. This micro-scale stock management would see both retailers and manufacturers incur considerable additional handling costs, and thus seems very implausible to us. It would also pose more explicit legal risks for the tobacco companies involved.

A limitation with this study is that New Zealand does not have detailed data on whether the seven different GHWs required on packaging during the time period of this study have a differential impact on smokers of the. However, after completing our analysis, an Australian report [7] described relevant data for GHWs that have similar or identical graphics and text warnings to the ones used in New Zealand (Table 2). The Australian survey results for unaided recall and smokers' beliefs about GHW effectiveness are consistent with our decision to describe the "corpse with toe-tag" image as "least disturbing" and to use it as the reference in our analysis (Table 1). Most of the other results in Table 2 also confirm our categorisation of the three "most disturbing" images. Indeed, these particular three GHWs were described in the Australian report as being the most "shocking" and the ones that "received high recall and most comment" (p24 [7]).

**Table 2:** Comparison of the graphic health warnings (GHWs) in this New Zealand (NZ) study with GHWs and survey data for similar GHWs in Australia

	**Theme of the GHW used in New Zealand (see Table 1)**						
	**Corpse with toe-tag**	**Pregnant woman & infant**	**Damaged heart**	**Damaged lungs**	**Gangrenous toes**	**Eye (blindness)**	**Diseased mouth/cancer**
***GHWs in NZ in this study***							
**Our *a priori *assessment used for this NZ study (see Table 1)**	Least disturbing	Moderately disturbing	Moderately disturbing	Moderately disturbing	Most disturbing	Most disturbing	Most disturbing
**Actual main warning text**	"Tobacco smoke is poisonous"	"You are not the only one smoking this cigarette"	"Smoking causes heart attacks"	"Over 80% of lung cancers are caused by smoking"	"Smoking causes gangrene"	"Smoking causes blindness"	"Smoking causes mouth cancer"

***Nearest equivalent GHWs in an Australian study (out of 14 different GHWs) ***[7]							
**Similarity of Australian graphic image to the NZ one**	Shows a container of toxic chemicals vs the NZ one of a foot of a corpse with a toe-tag	Shows a sick infant which is very similar to the NZ one (but the NZ one also has an image of a pregnant woman)	Shows a heart bypass operation vs the NZ one of a heart with blackened tissue from a heart attack	Shows a lung cancer vs the NZ one of a diseased versus a healthy lung	Identical photograph of foot with gangrene in both countries	Identical photograph of an eye in both countries	Identical photograph of a mouth in both countries
**Actual main warning text (Australian)**	"Tobacco smoke is toxic"	"Smoking harms unborn babies"	"Smoking causes heart disease"	"Smoking causes lung cancer"	"Smoking causes peripheral vascular disease"	"Smoking causes blindness"	"Smoking causes mouth and throat cancer"
Rank in terms of unaided recall by smokers*	13 = (lowest)	1 (highest)	5	2	3	8 =	4
Rank in terms of effectiveness in "discouraging people from smoking" according to *all *smokers**	12 = (lowest)	3 =	9 =	2	3 =	9 =	1 (highest)
Rank in terms of effectiveness in "discouraging people from smoking" according to *non-contemplative *smokers***	12 = (lowest)	3 =	10 =	3 =	2	7 =	1 (highest)

* See Table fifteen in the published report [7].

** See Table fifty one in the published report [7].

*** See Table fifty two in the published report [7].

In response to the findings of this study, we suggest governments should require tobacco companies to submit information on the print runs of the different GHWs. These reports should be tested against audits and regular field surveys, which would be required to validate the data submitted. Given the evidence that GHWs are an effective tobacco control intervention [8,9], and cost taxpayers nothing, such monitoring is important if GHWs are to have full impact on smokers. As a longer term and more comprehensive measure, the industry's apparent reluctance to comply with the new regulations strengthens arguments in favour of restructuring the tobacco market [10,11] so that a non-profit health agency, whose primary interest is public health not profit, controls all aspects of cigarette pack content and design.

## Competing interests

Although we do not consider this a competing interest, for the sake of full transparency we note that all of the authors (except JP and JS) have conducted work for health sector agencies on tobacco control issues.

## Authors' contributions

The study was designed by NW, GT, RE, and JH. Shopkeeper interviews were by JL. Collection of cigarette packs was undertaken and organised by all authors (except JL and JS). Data entry and statistical analyses was by JP, NW and JS. All authors participated in manuscript drafting and all authors read and approved the final manuscript.
